# Performance of ChatGPT on optometry and vision science exam questions

**DOI:** 10.1111/opo.13544

**Published:** 2025-07-09

**Authors:** Nayuta Yoshioka, Vanessa Honson, Revathy Mani, Sharon Oberstein, Kathleen Watt, Vinod Maseedupally

**Affiliations:** ^1^ School of Optometry and Vision Science UNSW Australia Sydney New South Wales Australia

**Keywords:** artificial intelligence, large language models, optometry assessment, optometry education

## Abstract

The rapid proliferation of Large Language Models (LLM) tools, such as ChatGPT developed by OpenAI, presents both a challenge and an opportunity for educators. While LLMs can generate convincing written responses across a wide range of academic fields, their capabilities vary noticeably across different models, fields and even sub‐fields. This paper aims to evaluate the capabilities of LLMs in the field of optometry and vision science by analysing the quality of the responses generated by ChatGPT using sample long answer questions covering different sub‐fields of optometry, namely binocular vision, clinical communication, dispensing and ocular pathology. It also seeks to explore the possibility of LLMs being used as virtual graders. The capabilities of ChatGPT were explored utilising various GPT models (GPT‐3.5, GPT‐4 and o1 models, from oldest to newest) by investigating the concordance between ChatGPT and a human grader. This was followed by benchmarking the performance of these GPT models to various sample questions in optometry and vision science. Statistical analyses include mixed‐effect analysis and the Friedman test, Wilcoxon signed‐rank test and thematic analysis. ChatGPT graders awarded higher marks compared to human graders, but significant only for GPT‐3.5 (*p* < 0.05). Benchmarking on sample questions demonstrated that all GPT models can generate satisfactory responses above the 50% ‘pass’ score in many cases (*p* < 0.05), albeit with the performance varying significantly across different sub‐fields (*p* < 0.0001) and models (*p* = 0.0003). Newer models significantly outperformed older models in most cases. The frequency of thematic response errors was more mixed between GPT‐3.5 and GPT‐4 models (*p* < 0.05 to *p* > 0.99), while o1 made no thematic errors. These findings indicate ChatGPT may impact learning and teaching practices in this field. The inconsistent performances across sub‐fields and additional implementation considerations, such as ethics and transparency, support a judicious adaptation of assessment practice and adoption of the technology in optometry and vision science education.

**Key points**

- Earlier models of ChatGPT (GPT‐3.5 and GPT‐4) demonstrated variable but generally passable performance across various optometry and vision science written response questions. The latest model (o1) excelled across all questions.
- ChatGPT showed potential as a grader of written questions. The scores awarded by ChatGPT graders were higher, but not significantly different to a human grader.
- The result of the study suggests there is an urgent need for optometry and vision science educators to adopt new learning and teaching strategies in the ‘ChatGPT‐era’.

## INTRODUCTION

Artificial Intelligence (AI) represents technologies that are designed to mimic human learning and reasoning to perform complex tasks. A Large Language Model (LLM) is a generative artificial intelligence (GenAI) capable of generating convincing text output via prediction of the subsequent series of text based on large text‐based training data (corpus)^1^, ^2^ and a ‘transformer’ architecture, which processes text input based on weighted relevance of different elements.^1^ LLMs, like GPT (Generative Pre‐trained Transformer, OpenAI Inc., openai.com/) are highly capable of completing tasks that were previously challenging for traditional AI models.^3^, ^4^ The capabilities of these models are evolving rapidly, as evident by the release and subsequent updates of ChatGPT™ (a chatbot service based on the GPT models) in late 2022. It initially featured GPT‐3.5 as the foundational model, followed rapidly by the release of GPT‐4 in early 2023^5^, ^6^, ^7^ and a new generation of reasoning models (o1 series) in late 2024.^8^ Each new model generation demonstrated significantly greater capabilities over previous models.^4^, ^8^ Furthermore, the cost of these models has dropped dramatically,^9^ and models with equivalent capacity continue to be released from other companies, including ‘open‐source’ (specifically, open‐weight) models, such as Llama™ (Meta AI, ai.meta.com/)^10^ and the DeepSeek™ series of models (Hangzhou DeepSeek Artificial Intelligence Basic Technology Research Co., Ltd., deepseek.com/en).^11^

The sudden proliferation and availability of highly capable and text‐generating tools pose a significant threat to academic integrity,^12^, ^13^ as they are reported to be capable of passing or even excelling in many tertiary or professional level written assessments.^4^, ^14^, ^15^, ^16^, ^17^ While concerns surrounding the erosion of *validity* in non‐invigilated written assessments via plagiarism and contract cheating are not new,^18^, ^19^ the ready availability of LLMs further exacerbates these concerns. Accordingly, it is important to address the immediate threat to academic integrity posed by LLMs in optometry and vision science education.

In contrast, LLMs are reportedly utilised within the classroom to improve student learning outcomes,^20^, ^21^, ^22^, ^23^, ^24^ and are tools that may both elevate teaching and learning practices and address educational inequity.^23^, ^25^ Further, LLMs have the potential to be utilised for assessing students' written work given their capability to contextualise and analyse complex text data.^26^, ^27^, ^28^ However, the concept of automated assessment is not without ethical dilemmas and concerns surrounding accountability.^29^, ^30^ Outside of the educational setting, GenAI have the potential to improve clinical outcomes within the medical and healthcare field.^16^, ^17^, ^31^, ^32^, ^33^, ^34^, ^35^, ^36^, ^37^

Given the complex potential impact of LLMs, educators and professionals must be careful not to engage in counter‐productive discourse that is dismissive or resistant to change.^25^ Instead, discussions around utilisation and integration of GenAI in optometric teaching, learning and clinical workflow are required.^38^ This process requires understanding of the capabilities of LLMs within the field of optometry and vision science.

**FIGURE 1:**
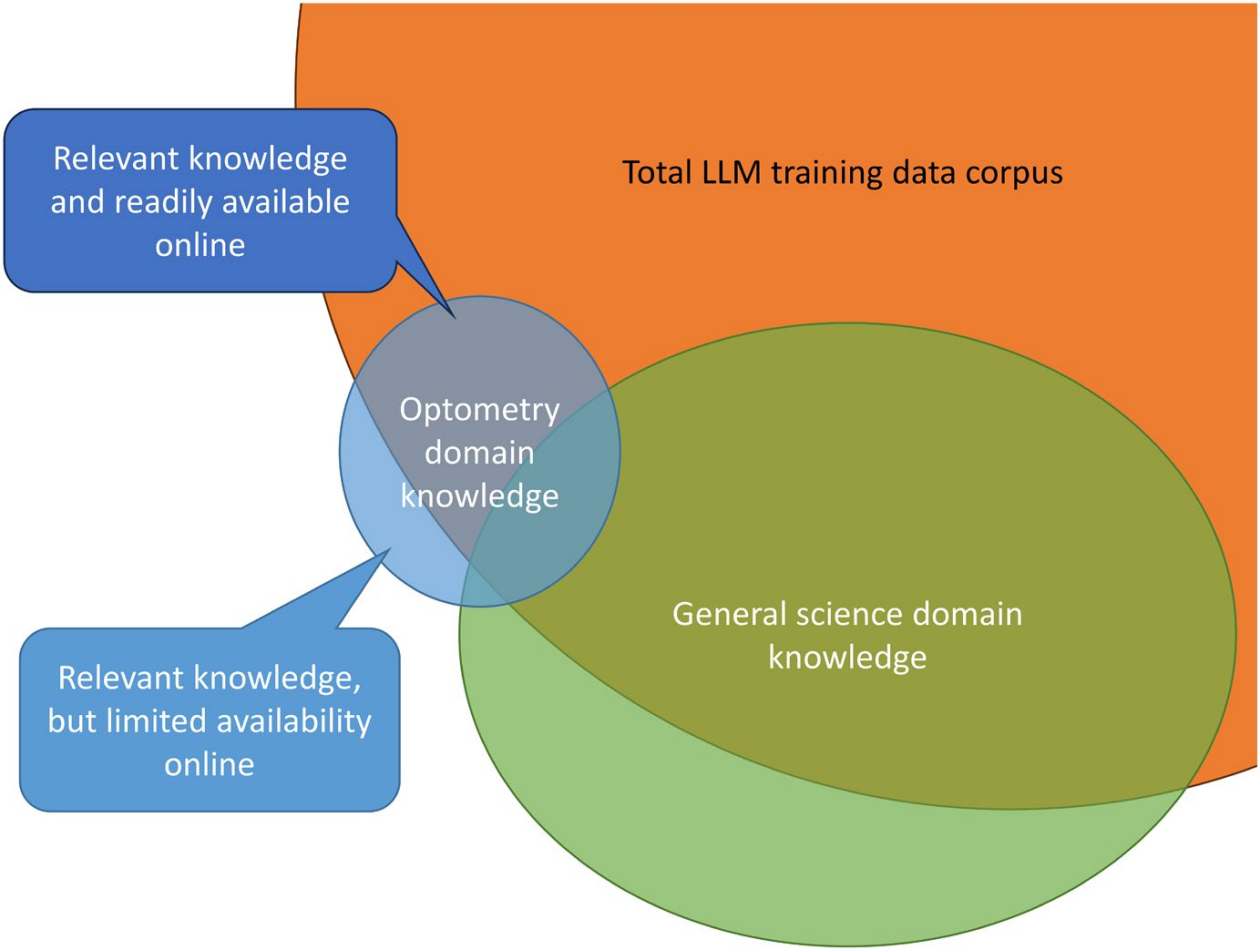
A representative Venn diagram of domain specificity. These show the potential overlap and absence/under‐representation of data included within a Large Language Model's (LLM) pre‐training data (orange oval) versus relevant corpus of data in different domains (green: general science, blue: Optometry and vision science). Note that domain knowledge not covered by the LLM corpus does not necessarily mean such data are absent from the pre‐training data altogether, but may be under‐represented due to the limited volume of high‐quality text data within the corpus.

Firstly, the capability of LLMs may vary significantly by the model. For example, GPT‐4 shows substantially higher capabilities for most domains than GPT‐3.5.^4^ In turn, OpenAI's new reasoning model, o1, demonstrated improved capabilities over GPT‐4.^39^ This observation is generalisable to the field of ophthalmology, with later models overall showing greater capabilities.^17^, ^31^, ^40^, ^41^ In spite of this, predicting the capabilities of an LLM for a specific task remains challenging,^3^, ^42^ especially as their capabilities are expected to vary across different fields and disciplines, following the concept of *domain specificity*.^2^, ^17^, ^23^, ^43^ In this paper, the term *domain* is used to refer to specific fields and disciplines, such as optometry and vision science. While not the sole determining factor, the performance of an LLM is affected by the quantity and quality of the training data—the greater the quantity and quality, the better the performance of the LLM.^3^ However, not all training data may be relevant to a given domain, and one cannot necessarily infer the performance of an LLM for a specific domain based on the total pre‐training corpus size and content.^2^, ^23^, ^42^, ^43^ GPT models are trained mostly on publicly available internet text data, although the exact dataset composition and training processes are not available due to competitive and safety implications as stated by OpenAI.^4^ The amount of text data in a niche domain, (such as optometry and vision science) is predicted to be smaller than for a more mainstream domain, such as general science (Figure 1). Note that domain specificity is likely to be complicated further by the intersections of different disciplines. For instance, overlaps between optometry and science, medicine and/or ophthalmology occur (Figure 1). This suggests that even within the domain of optometry and vision science, LLM performance is likely to vary across sub‐domains. This has recently been observed in a report of specific sub‐domains in which the newer and more capable o1 model performed worse than older models.^17^ This ‘jagged’ capability of LLMs across different domains and sub‐domains is affirmed within OpenAI's technical report^4^ and discussed in great detail in recent research studies.^14^, ^15^, ^42^

Additionally, LLMs are susceptible to biases due to their pre‐training corpus and training processes.^23^ LLMs' outputs exhibit biases towards knowledge and tasks that are weighted more heavily within the corpus. Specifically, given the corpus is predominantly text data from the internet, the LLM's response is biased by how visible or ‘popular’ the knowledge or tasks are on the internet.^42^, ^44^, ^45^ Due to the lack of information on the pre‐training corpus and the actual training process, this irregularity in capability is difficult to predict.^42^ Thus, LLM capabilities should be explored for each domain/discipline and even each specific task.

Therefore, the overarching aim of the paper was to scope the capabilities of LLMs, such as ChatGPT, within the domain of optometry and vision science to provide guidance to educators within this field. Firstly, one of the potential applications of LLM within the educational field is as a virtual marker. While ethical concerns exist with regard to automated marking of written assessments,^29^, ^30^ the capability of LLMs for grading assessment items within this domain needs to be investigated. This will allow optometry and vision science educators to make informed decisions regarding teaching and learning strategies in the ‘ChatGPT‐era’. Therefore, the first specific aim of the study was to evaluate the concordance between ChatGPT's grading of responses to optometry and vision science written questions with that of a human assessor.

Further, there is a need to explore the capabilities of ChatGPT on various written assessment questions within an optometry and vision science programme to inform assessment designs. Thus, the second aim of the study was to compare the proficiency of ChatGPT in generating a response to a range of written questions pertinent to optometry and vision science when utilising different generations of the model, namely GPT‐3.5, GPT‐4 and o1 models.

These will be addressed individually through two separate studies as discussed in the Methods section.

## METHODS

### Instrumentation

ChatGPT by OpenAI was chosen as the model to be tested based on the availability and capability of various models at the time of the experiment (February 2023–January 2025). ChatGPT powered by GPT‐3.5 was accessible for free via the OpenAI website (chat.openai.com/) until mid‐2024. ChatGPT powered by GPT‐4 was accessed either via Poe (Quora Inc., poe.com/, which offered limited access to GPT‐4 in early 2023) or from ChatGPT Plus,^46^ a paid subscription service for ChatGPT. The o1 model was accessed via ChatGPT Enterprise in January 2025. Each response to the question or grading of an answer was generated using a new instance of the chat. Where possible, custom instructions, plug‐ins, saving of chat history and allowing the chat log to be utilised for training purposes were disabled. GPT/ChatGPT were constantly updated during the study, and thus, the versions (when available) are detailed under individual sections.

All statistical analyses and graph generation were conducted using GraphPad Prism v10.4.1 (GraphPad Software, graphpad.com) unless otherwise indicated. The tests utilised are discussed under the respective sections below. The study used only publicly available or non‐confidential information and responses generated from ChatGPT. The example questions utilised for this study have been made available publicly on UNSWorks under CC‐BY licence.^47^ No personal or sensitive information such as past examination responses from students were included. Grading and evaluation of the LLMs' responses were conducted by the study authors or by the LLMs. No additional human participants were required, apart from the paper authors, who graded the responses. Accordingly, there was no requirement to seek ethical approval for the study.

### Test questions

Sample optometry and vision science written response questions (including past assessment questions, example questions and in‐class questions for Problem Based Learning activities) covering a range of topics were collated. Given that OpenAI stored all ChatGPT inputs and may utilise them for the purpose of training their models,^5^ only questions that were not planned to be used for summative assessment purposes were chosen. These were subsequently released publicly on UNSWorks.^47^

**FIGURE 2:**
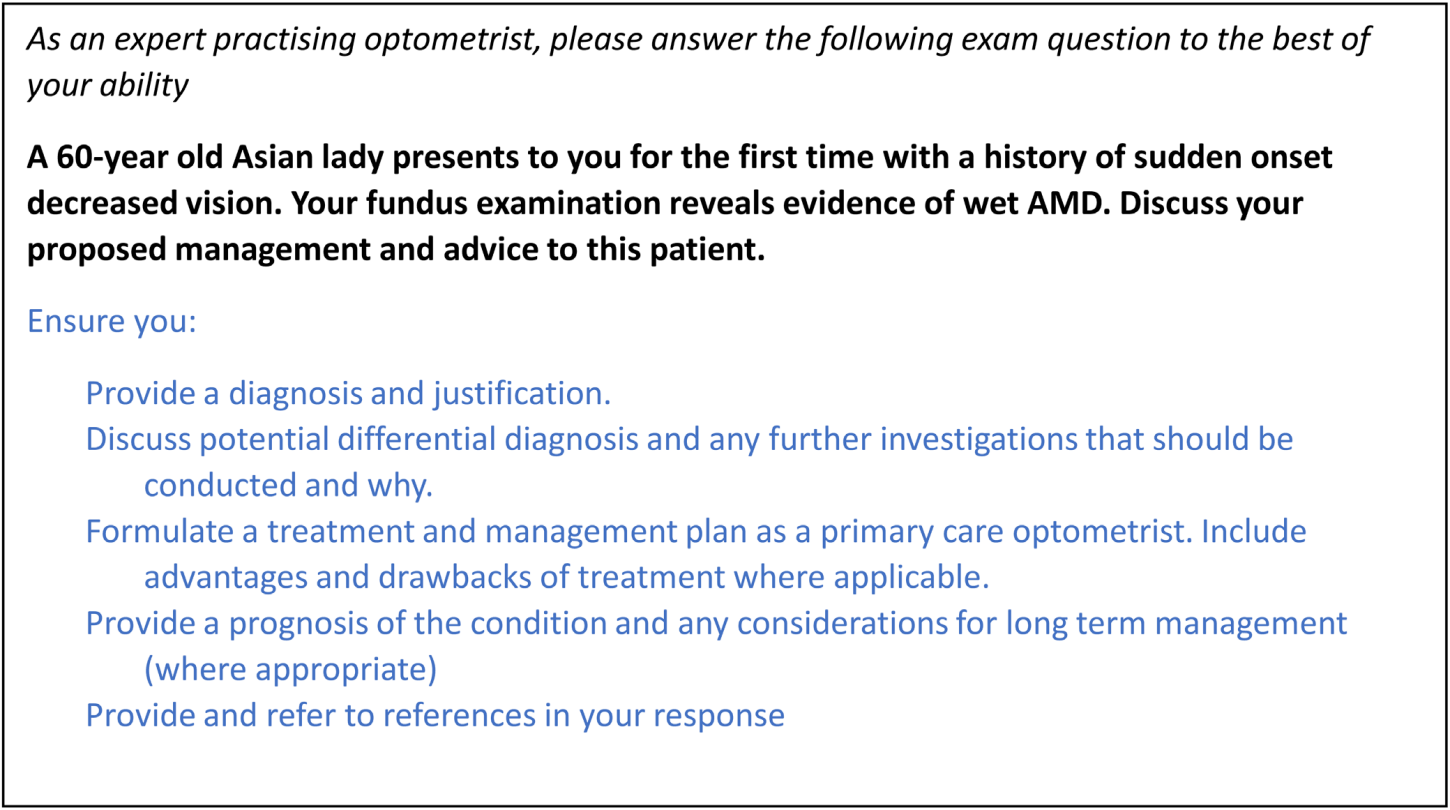
An example of how the questions were provided to ChatGPT. The first sentence (shown in italic) was a common prompt used for all questions to provide broadly the context and expectation of the response. This was followed by the body of the question, shown in bold. The part in blue font was not required for all questions—it was only provided to prompt ChatGPT to perform part of the task that was understood implicitly by the students. AMD, age‐related macular degeneration.

LLM's performance is sensitive to how the user's instruction (*prompt*s) are structured and the presence/absence of any additional information provided. However, the optimal method of ‘prompt engineering’ is still developing.^2^, ^8^ Therefore, standardised prompts accompanied the questions when entered into ChatGPT. The prompt was authored to reflect how a knowledgeable user might prompt the system. Only zero‐shot prompts were investigated for consistency (Figure 2).

Questions were utilised in their original form as much as possible. Modifications were conducted only to provide ChatGPT with context and instructions that are expected to be understood implicitly by students, such as the need to provide and discuss differential diagnoses in an ocular disease case study (Figure 2).

An outline of the questions utilised is detailed in Table 1 and includes ophthalmic dispensing, patient communication, binocular vision, advanced ocular imaging knowledge and an ocular disease case study. These are coded D1, C1, B1, P1 and P2, respectively.

**TABLE 1:** List of optometric questions from different sub‐domains, description of the tasks and corresponding Bloom's Taxonomy Level. Full information of the questions and answers available from UNSWorks.^47^

Question code and sub‐domain	Summary of task	Skills assessed	Bloom's taxonomy level
D1: Dispensing	Evaluate the patient case to identify and justify the selection of specific frames and lenses that best meet their visual and lifestyle needs	Frame selection, lens selection, discussion	Level 4 to Level 5 ‘Analyse’, ‘Evaluate’
C1: Clinical communication	Calculation and analysis of far and near point. Appraising appropriateness of history and symptoms questions related to this area	History taking, calculations, discussion	Level 4 to Level 5 ‘Analyse’, ‘Evaluate’
B1: Binocular vision	Calculate and evaluate clinical parameters for diagnosis of binocular vision conditions	Calculations, analyse and diagnose, case report	Level 4 to Level 5 ‘Analyse’, ‘Evaluate’
P1: Ocular pathology (imaging)	Explain the diagnostic complications for glaucoma in high myopes using various imaging techniques, including OCT	Knowledge of latest literature on OCT, ocular disease and myopia	Level 2 ‘Understand’
P2: Ocular pathology (Dx and Mx)	Evaluate the case study for an elderly Asian lady presenting with wet AMD‐like changes and justify the most likely diagnosis	Diagnosis, prognosticating, discussion	Level 4 to Level 5 ‘Analyse’, ‘Evaluate’

Abbreviations: AMD, age‐related macular degeneration; OCT, optical coherence tomography.

### Study phases

To investigate various capabilities of ChatGPT in the domain of optometry and vision science teaching and learning, two experiments were conducted to address the two aims discussed in the Introduction.

#### Study 1: ChatGPT as a virtual marker

Eight responses were generated for an existing ophthalmic dispensing case study (D1, Table 1) from the second‐year curriculum of the University of New South Wales (UNSW)'s Bachelor of Vision Science/Master of Clinical Optometry programme, using GPT‐3.5 (Feb 13 Version accessed on 13th February 2023, OpenAI Inc., chat.openai.com/). These included the practical application of prescribing and fitting corrective spectacle lenses to improve a patient's vision. These eight ChatGPT responses were then marked by a human grader (NY) and ChatGPT utilising different models (GPT‐3.5, GPT‐4, o1). For the first attempt, ChatGPT with GPT‐3.5 (13 March 2023 version) was provided with each of the eight responses accompanied by the marking rubric and an instruction to grade the response in accordance with the rubric. To determine the capability across models and version, this marking was reattempted using GPT‐4 Turbo (accessed 16 January 2024) and o1 (accessed 27–28 January 2025). The scores awarded to the responses by the human assessor and the virtual markers were analysed using Friedman's test with Dunn's multiple comparison test. Given the small sample sizes (eight responses graded), a non‐parametric approach was deemed more appropriate.

#### Study 2: Benchmarking ChatGPT's responses to optometry and vision science questions

**FIGURE 3:**
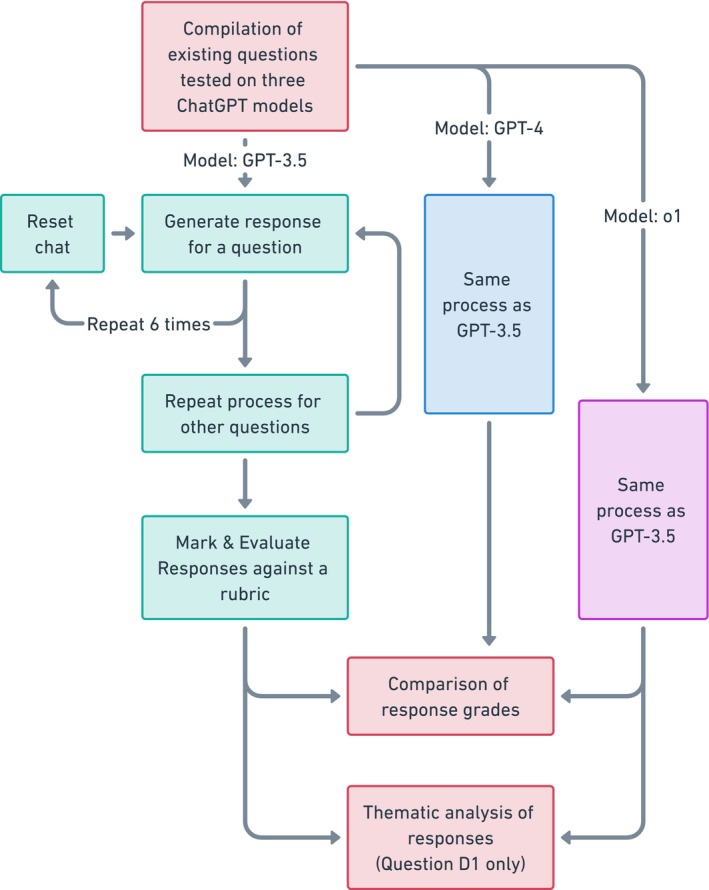
Flow chart for demonstrating the process of evaluating the capabilities of GPT‐3.5, GPT‐4 and o1 for answering optometry and vision science questions from different sub‐disciplines. These included B1: Binocular Vision, C1: Clinical Communication, D1: Dispensing, P1: Ocular Pathology (Imaging) and P2: Ocular Pathology (Diagnosis and Management) (see Table 1).

The capabilities of various ChatGPT models (GPT‐3.5, GPT‐4 and o1) in answering questions from various optometry and vision science subfields were investigated (Table 1). Between six and ten responses were generated using the three models, respectively, for each question, scored by a human grader and analysed as shown in Figure 3. Question D1 utilised the initial eight GPT‐3.5 responses as discussed in Study 1 plus five additional responses (Mar 13 Version accessed on 13 March 2023, OpenAI Inc., chat.openai.com/) for a total of 13 responses, as well as six responses generated by GPT‐4 (accessed via Poe, Quora Inc., poe.com/, 20 March 2023). All other questions utilised GPT‐3.5 and GPT‐4 accessed between 24 July and 30 August 2023 (20 July or 3 August versions). For testing the o1 model, ChatGPT with o1 was accessed between 17 and 24 January 2025. With the exception of question C1, all responses were marked by a single marker (NY) in accordance with a marking rubric. C1 responses were marked by the author of the question using the marking rubric (VH). All grades were converted to percentages to allow comparison between different question types.

The normality for the scores awarded to each response set (questions × models) was assessed using the Shapiro–Wilk test and violations were observed in multiple response sets (*p* < 0.05). Given the small sample sizes and the presence of non‐normal distributions, a non‐parametric approach was deemed more appropriate. The median of resultant grades for each question by each GPT model was compared against a ‘pass’ grade of 50% with a one‐sample Wilcoxon signed‐rank test. To determine the performance of different GPT models for answering various questions, the result was analysed using a mixed‐effect model with Geisser–Greenhouse correction and Tukey's multiple comparison test.

Thematic analysis was also conducted for question D1 to determine the type of errors made within the written responses. Thirteen responses from GPT‐3.5, six responses from GPT‐4 and o1 each were compared. The type of errors analysed included contextual errors (e.g., recommending a solid tint for a spectacle to be worn constantly), irrelevant responses (e.g., a blue light blocking lens for a patient with limited digital device use), extracurricular content (any content not taught) and for basic misunderstanding. The frequency of such errors was analysed using Fisher's exact test.

## RESULTS

### Study 1: ChatGPT as a virtual grader

**FIGURE 4:**
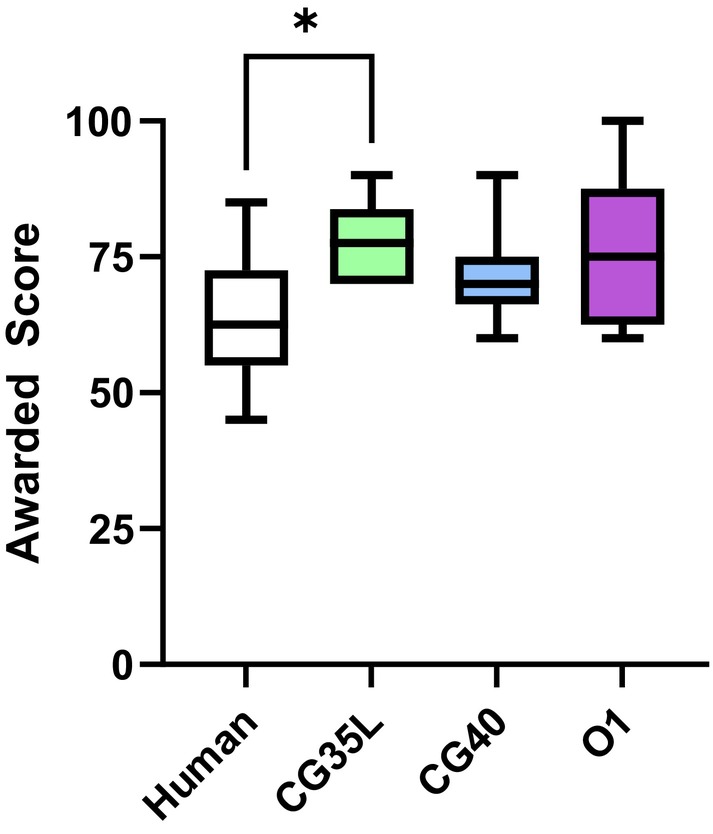
Comparison of the grade awarded to the ChatGPT generated responses for a dispensing question (D1) by ChatGPT‐3.5 Legacy model (CG35L), ChatGPT‐4 (CG40) and ChatGPT‐o1 versus an experienced human grader. *p < 0.05, Dunn's multiple comparisons test against ‘Human’.

**FIGURE 5:**
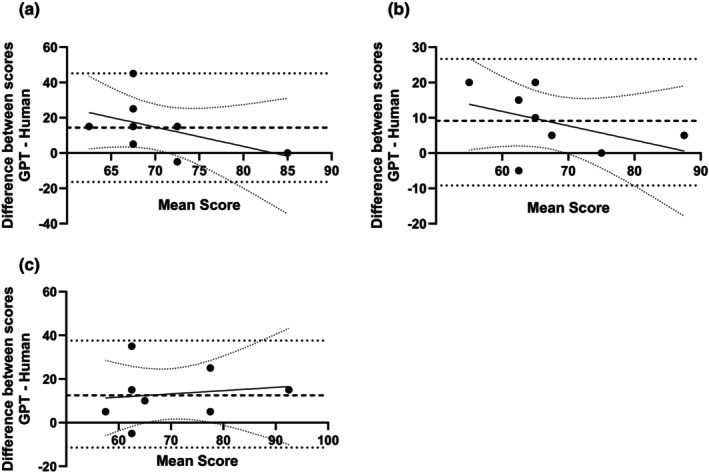
(a) Bland–Altman plot comparing the grade awarded to the response by GPT‐3.5 and a human grader. The central dashed line indicates the mean difference in grade between the two markers (GPT minus human) and the dotted lines indicate the upper and lower 95% limits of agreement. The solid line indicates the proportional bias line and the fine dotted lines represent the 95% confidence interval. (b) Bland–Altman plot comparing the grade awarded to the response by GPT‐4 grader and a human grader. The central dashed line indicates the mean difference in grade between the two markers (GPT minus human) and the dotted lines indicate the upper and lower 95% limits of agreement. The solid line indicates the proportional bias line and the fine dotted lines represent the 95% confidence interval. (c) Bland–Altman plot comparing the grade awarded to the response by o1 grader and a human grader. The central dashed line indicates the mean difference in grade between the two markers (GPT minus human) and the dotted lines indicate the upper and lower 95% limits of agreement. The solid line indicates the proportional bias line and the fine dotted lines represent the 95% confidence interval.

Statistics comparing the grade awarded to the responses by the human grader and the three ChatGPT models are shown in Table 2. All models awarded higher grades to the responses compared with the human marker, but the difference was significant only for GPT‐3.5 (Figure 4, *p* = 0.04, Dunn's multiple comparison test). A Bland–Altman analysis was conducted to analyse further the nature of the discrepancy between the human and AI graders (Figure 5a–c). While the human and GPTs showed general agreement on the grade for the higher quality responses, the difference between the two grading agents increased with lower quality responses. Thus, ChatGPT demonstrated non‐significant discrepancies when scoring examination responses, compared with human markers.

**TABLE 2:** Comparison of scores awarded to a response for a dispensing sample question by human graders and ChatGPT grader (utilising GPT‐3.5, GPT‐4 and o1 models).

Grader	Median	IQR	Minimum	Maximum	*p*‐Value
Human	62.5	17.50	45	85	–
GPT3.5	77.5	13.75	70	90	0.04*
GPT‐4	70.0	8.75	60	90	0.36
O1	75.0	25.00	60	100	0.10

* *p* < 0.05 compared against human grader, IQR, interquartile range. Wilcoxon signed‐rank test.

### Study 2: Benchmarking ChatGPT's responses to optometry and vision science questions

In general, ChatGPT performed well in answering the optometry and vision science questions, with the median mark significantly above the pass grade of 50% in many instances (Table 3, one‐sample Wilcoxon signed‐rank test, *p* < 0.05). A notable exception to the overall passable performance by ChatGPT was GPT‐3.5 and GPT‐4 answering the Clinical Communication (C1) and the Pathology Imaging questions (P1), as well as GPT‐3.5 answering the Pathology Imaging questions (P1), where the median scores were not significantly above the pass grade.

**TABLE 3:** Comparison of median performance for ChatGPT (using the models GPT‐3.5 [CG35], GPT‐4 [CG40] and o1 ) when responding to different question types.

	Model	Median	IQR	Minimum	Maximum
Dispensing (D1)	CG35	65**	15.0	45	85
	CG40	77.5*	12.5	60	80
	o1	100*	2.50	90	100
Clinical communication (C1)	CG35	35.7	3.57	35.7	50
	CG40	69.6	10.71	50	71.4
	o1	86.7*	8.33	80	93.3
Binocular vision (B1)	CG35	67.3*	9.13	56.3	70
	CG40	72.9*	5.92	59.8	74.8
	o1	85.3*	6.43	81.2	93.6
Pathology: Imaging (P1)	CG35	57.1	16.1	28.6	71.4
	CG40	57.1	21.4	42.9	71.4
	o1	89.3*	28.6	71.4	100
Pathology: Diagnosis and management (P2)	CG35	58.8	15.0	45.9	63.5
	CG40	75.3*	5.88	72.9	82.4
	o1	89.4*	6.77	83.5	92.9

Abbreviation: IQR, interquartile range.

*p < 0.05, **p < 0.01 Wilcoxon signed‐rank test (two‐tailed), testing the alternative hypothesis that μ ≠ 50%.

**FIGURE 6:**
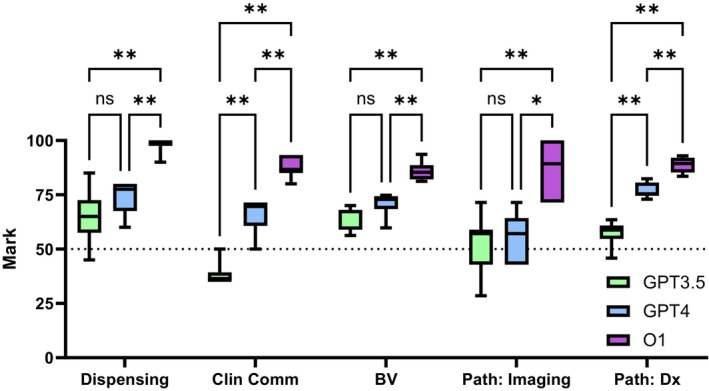
Performance of GPT‐3.5, GPT‐4 and o1 for various subdomain tasks within optometry. The newer models demonstrated significantly better capabilities in most cases (Tukey's multiple comparison, *p < 0.05, **p < 0.01). BV, binocular vision; Clin Comm, clinical communication; Path, pathology; Path imaging, pathological imaging.

Across all question types and models, the median score for each question ranged from 35.7 to 100, demonstrating a wide range of question types and models. A mixed‐effect analysis showed significant main effects of question type (*F*(4, 36) = 11.65, *p* < 0.0001) and the model used (*F*(1.961, 52.95) = 143.7, *p* < 0.0001), as well as the interaction between the question and the model (*F*(8, 54) = 4.541, *p* = 0.0003). Analysis of the effect of the models on Tukey's post‐hoc test illustrate that in almost all cases, newer GPT models outperformed older models significantly (*p* < 0.05, Figure 6 and Table S1) except for questions D1, B1 and P1 when comparing GPT‐3.5 to GPT‐4. The O1 model outperformed both models for all questions (*p* < 0.05, Table S1). Tukey's post‐hoc analysis further revealed that each model demonstrated non‐uniform capabilities when answering different types of optometric questions (Table S1), with GPT‐3.5 performing worse for C1 against all other tests except P1 (*p* < 0.01); GPT‐4 performed worse for P1 against all tests except C1 (*p* < 0.01) and o1 demonstrating significantly higher performance with D1 against all other question types except P1 (*p* < 0.05). However, it is worth noting that o1 showed a more consistent performance across the board. The median finding (IQR) when all scores were aggregated across questions were 57.65 (45.44–66.05) for GPT‐3.5, 71.43 (59.16–75.07) for GPT‐4 and 89.41 (85.46–95.19) for o1.

The type of thematic errors made by ChatGPT are shown in Table 4. The O1 result was not analysed as five out of the six responses attained a full score and no major thematic errors were made by this model. While GPT‐3.5 appeared to make a greater number of thematic errors compared with GPT‐4, this difference was not significant (Table 4a, *p* = 0.15, Fisher's exact test). The most frequent type of errors made by both GPT‐3.5 and GPT‐4 were contextual errors. While again the frequency of this error was not significantly different between the models (Table 4b, *p* = 0.16, Fisher's exact test), two distinct errors were observed. Firstly, based on recommending a solid tint for constant wear spectacles (contextual error, tint), and secondly, recommending a high index lens over a CR‐39/polycarbonate lens for a patient with low ametropia requiring lightweight spectacles (contextual error, refractive index). When taken in isolation, the contextual error for tint was significant, (*p* = 0.05, Table 4c), but not significant for the refractive index error (*p* > 0.99, Table 4d). This highlights that while there may be patterns in the tasks, LLMs may still excel or struggle, and this may be difficult to predict.

**TABLE 4:** (a) Contingency table showing the frequency of errors made per response for ChatGPT3.5 and GduePT4 (*p* = 0.15, Fisher's exact test). Results for o1 are not shown as no noteworthy thematic errors were made. (b) Contingency table showing the frequency of contextual errors made per response for ChatGPT3.5 and GPT4 (*p* = 0.16, Fisher's exact test). (c) Contingency table for the contextual error based on recommending a solid tint for constant wear spectacles. *The presence of this error type differed significantly between GPT3.5 and GPT‐4 (*p* = 0.05, Fisher's exact test). (d) Contingency table for the contextual error based on recommending a high refractive index lens over a CR‐39/polycarbonate lens for a low ametrope requiring a light weight frame. Differences in the frequency of this error type were not statistically significant (*p* > 0.99, Fisher's exact test).

(a)					
Models	Total number of errors				Total
	1	2	3	4	
GPT3.5	2	7	2	2	13
GPT‐4	4	1	1	0	6
Total	6	8	3	2	19

(b)				
Models	Contextual error (combined)			Total
	0	1	2	
GPT3.5	2	4	7	13
GPT‐4	0	5	1	6
Total	2	9	8	19

(c)			
Models	Contextual error (tint)		Total
	Absent	Present	
GPT3.5	4	9	13
GPT‐4	5	1	6
Total	9	10	19

(d)			
Models	Contextual error (index)		Total
	Absent	Present	
GPT3.5	4	9	13
GPT‐4	0	6	6
Total	4	15	19

## DISCUSSION

### Study 1: LLM as an assessment grader

LLMs have the potential to improve learning and teaching processes as a virtual tutor or to enhance learning activities has been highlighted previously.^20^, ^21^, ^22^, ^23^, ^24^ When marking optometry and vision science question responses, the results for LLM grading did not show significant disparity from human graders with the newer models of GPT (GPT‐4 and o1, Figure 4). However, LLMs generally awarded higher grades for the responses when compared with human graders. These findings are consistent with previous works from other domains, which also found significant agreement between instructor and LLMs' assessment of student responses, but with LLMs often awarding higher marks.^26^, ^28^ A potential source of disparity is the training process of these models. One of the aims of many companies working on generative AI is to ensure that the models' responses attain ‘alignment’ with human needs.^2^ In other words, minimising the chance of harm to human users. As a result, it may be less willing to award low marks, even for poor responses as these may negatively impact the student.

Thus, it is proposed that AI graders be used with caution. While current LLMs have low rates of ‘hallucination’ (i.e., factually erroneous responses that are presented plausibly and convincingly),^48^ there are ethical hurdles to be overcome before educators can trust LLMs with important tasks, such as the grading of summative, high stakes assessments. Areas of ethical concern include, but are not limited to, the validity and fairness of the tool (both actual and perceived), transparency of the marking procedure, perceived dehumanisation and anxiety surrounding the adoption of technology.^27^, ^29^, ^30^ While other roles for LLMs in assessment grading are possible, such as assistance for human graders and writing feedback, the ethical and legal hurdles of providing student data to a third party (i.e., the provider of the LLM service) currently exists, and educational institutes need to adopt these technologies with caution.

### Study 2: LLMs may perform well in written optometry and vision science questions

The present results show that LLMs have the potential to answer a wide range of optometry and vision science questions. While ChatGPT responses generally passed, the grades were non‐uniform (Table 3) even within a discipline, and this was complicated further by differences in performance between LLM models.^23^, ^24^, ^42^

As expected, GPT‐4 outperformed GPT‐3.5, while in turn, o1 outperformed GPT‐4 (Table 3 and Figure 6). With the exception of Pathology Imaging (P1), all questions were considered to be Level 4 to Level 5 on the Bloom's Taxonomy scale, as these required ‘Analysis’ and ‘Evaluation’ of clinical information—see Table 1.^49^ For clinical case study tasks that required critical analysis skills, ChatGPT‐4 outperformed ChatGPT‐3.5. In turn, when comparing o1 to ChatGPT‐4, o1 showed significantly higher performance across all questions. Such observations are in line with other reports examining the ability of LLMs to answer various tests in fields both within and outside the fields of optometry/ophthalmology.^14^, ^15^, ^17^, ^24^, ^41^, ^50^

It should also be noted that the score awarded may be lower with different marking contexts and expectations. To make the marking process as objective as possible, the rubrics used did not allow for grades to be awarded/deducted based on the overall impression of the response or for the deduction of points for an incorrect or irrelevant statement. Thus, even if responses contained grossly incorrect statements, for example, responses to D1 (Table 4), no points were deducted. Further, the context of the question is important. For instance, with the pathology case study question (P2), the most likely diagnosis for the case was polypoidal choroidal vasculopathy (PCV), based on the ethnicity of the patient, with wet age‐related macular degeneration (AMD) being the primary alternative diagnosis.^51^, ^52^ While the clinical management of PCV and AMD may differ in a clinical ophthalmology context, the recommended management for primary eye care practitioners of these two conditions is very similar. Thus, even if the less correct diagnosis of wet AMD was made, good grades were still awarded as long as the management was correct. On the other hand, if the same question was provided to ophthalmology students, then the overall score should be lower if AMD was the primary diagnosis. While all responses from o1 correctly identified wet AMD and PCV as the most likely diagnosis/primary differential diagnosis, both GPT‐3.5 and 4 generally failed to identify PCV as a potential diagnosis, except in one response from GPT‐4. The likely explanation for this is LLM's inherent bias to prefer responses that are more frequently represented in their corpus of data.^44^ In this case, AMD is more ‘internet famous’ than PCV, with approximately 291,000,000 hits versus approximately 34,400 hits for PCV on Google (google.com/, search date, 10 February 2025, search term [‘Age‐related Macular Degeneration’ OR ‘AMD’] and [‘Polypoidal Choroidal Vasculopathy’ or ‘PCV’]). While the exact composition of the corpus of data that GPTs have been trained on is not publicly available,^4^ a significant portion comprises publicly available internet data. Thus, this should provide an indication of the representation of these two diseases within the pre‐training data.

### Limitations and future directions

Limitations and challenges for exploring LLMs are innate to their rapidly evolving nature, which is reflected in the frequent changes in the models and the body of literature cited. Updates to the model and the addition of new features occurred frequently during the present study period. To minimise the impact of the GPT models being trained by the outputs of this study, all ChatGPT responses were collected with the option to use the chat output for training the model disabled whenever possible. If this option was not available, then the responses were simultaneously collected by 4–6 investigators in order to reduce the opportunity for the model to be trained on the output. Furthermore, as LLMs are not fundamentally designed to recall specific information,^2^ and due to the large absolute size of the training corpus, the impact of training on the present question set was expected to be non‐existent.

The field of AI and LLM evolves rapidly, and many foundational studies, including some of the most widely cited and impactful works,^1^, ^3^ are disseminated through online open‐access preprint servers such as *arXiv*, before^1^ or without^3^ traditional peer review. While the authors prioritised peer‐reviewed literature wherever possible, especially in discussions relating to educational applications and clinical relevance, it was necessary to reference preprints to provide the most up‐to‐date and accurate technical context. Future studies may benefit from revisiting these findings as more AI‐related research undergoes formal peer review when comparing student to ChatGPT responses.

A unique characteristic of LLMs is that the responses are variable.^2^ Within this study, substantial variabilities in the quality of the graded responses were observed, even when the same model graded the same response. Similarly, human graders are also known to exhibit significant variability when grading written responses.^53^ A limitation of this study is that it only employed a single human grader, and thus, it was not possible to compare the variability of the LLM graders to human graders. Future investigations could further compare the inter‐ and intra‐grader variability of these two types of graders.

Further investigations could also benchmark against student responses and expand the sub‐domains to be assessed. While benchmarking LLM performance against student responses to these questions would provide further insight, due to ethical concerns, these could not be analysed. The present study suggests that the latest generation of reasoning model (o1) is already reaching performance saturation. Thus, follow‐up studies could expand the investigation into additional sub‐domains, such as low vision and contact lenses, as well as benchmarking against more diverse question types, especially tasks which current generations of LLM are less capable of answering, such as image based and calculation questions.^15^

Finally, future investigations could benchmark and contrast the capabilities of various LLM foundational models. The current study's scope was to explore the potential use and limitation of different ‘generations’ of LLMs in optometry and vision science education. While exploration of different LLM models will be insightful, given the rapid development of this field, comprehensive comparison of different models remains outside the scope of this study. However, further investigations will be particularly insightful, especially if exploring open‐weight models (Llama™ by Meta^10^ and DeepSeek™^11^, ^54^), as these types of models become more accessible and prolific.

## CONCLUSION

The results of the study have shown that LLMs are able to generate satisfactory responses to various assessment questions in the field of optometry and vision science, and in many cases excel at these. Subsequent models showed significantly greater capabilities over preceding models. It should be noted, however, that even within this domain, LLMs showed uneven capabilities between different sub‐domains, albeit the overall capability of these models continues to improve with each new version. The proliferation of GenAI tools capable of producing satisfactory assessment responses with minimal user interaction presents a challenge to optometry and vision science educators. This is exacerbated further by the rapid reduction in the cost,^9^ and thus greater availability as well as the proliferation of open‐weight models.^10^, ^11^, ^54^ Therefore, there is an urgent need to review and redesign current assessment practices.^25^ Fortunately, recently published recommendations^15^, ^55^ for designing assessments in the ‘ChatGPT‐era’ appear to be compatible with existing contemporary pedagogical practices.^18^, ^19^ Notably, these frameworks advocate a shift away from utilising the final ‘product’ as the dominant evidence of student learning, for example, their written responses within their end of term essay and written examination. Instead, it is recommended to seek broader evidence via multiple and diverse assessment types, with emphasis on demonstrating the ‘process’ of learning, rather than the ‘product’. In the ‘ChatGPT‐era’ context, some of these assessments may allow or even integrate the use of GenAI. Simultaneously, there needs to be opportunities for students to be able to demonstrate their learning without GenAI assistance, especially in key skills within the profession.^55^ Thus, optometry and vision science educators need to consider what skills and learning are appropriate for AI integration when designing their assessments.

GenAI presents numerous possibilities for enhancing educational delivery,^23^, ^25^ such as automated assessment grading and feedback.^26^, ^27^ The current results have shown no significant difference between the score awarded by human and LLM graders, but a trend of LLM graders awarding higher scores was noted. Combined with the ethical, privacy and data handling considerations, optometric educational institutes need to adopt a cautious approach if integrating GenAI into the classroom setting in the current landscape at all levels, from institutional level to the instructor level.^56^

A significant risk associated with GenAI use in the educational setting is the risk of hallucination. While current LLMs are reported to offer low hallucination rates,^48^ it is still critical to acknowledge that errors do occur, as corroborated by these results (Table 4). Further, bias can be introduced within an AI model at various levels, from the pre‐training data to alignment training, either intentionally or unintentionally.^2^, ^57^ Educators and learners need to be mindful and educated of such risks and limitations when utilising these tools within educational settings. To minimise bias and factual errors in LLM‐generated responses, users must review AI outputs critically, and incorporate validation steps prior to their use in teaching or assessment contexts.^58^ Further, it is essential for educators to equip students to be able to utilise AI tools ethically and responsibly.^55^, ^56^

Many commercial LLM services rely on a third‐party provider, requiring users to store data with these services, thereby presenting cyber security risks around privacy and ethical data handling. While such concerns can generally fit within existing cyber security frameworks, LLMs present some additional risks.^15^, ^57^ The data provided to LLM services may be used for training and fine‐tuning AI models, which may fall outside the approved use of data.^57^ Furthermore, given LLMs' ability to produce responses with convincingly human‐like traits, users may misattribute a sense of trust to the AI chatbot. Thus, the user is at risk of overlooking appropriate cyber security protocols, leading to unintended information disclosure.^59^, ^60^ Therefore, it is crucial that users do not misattribute AI tools' trust and capabilities, and to be mindful of appropriate cyber security protocol at all times. Appropriate training in ethical and secure LLM use may help address such concerns.^56^, ^57^

Given the risks posed by GenAI, such as those discussed above, educational institutions need to conduct risk assessments of such tools and implement adequate guardrails. They need to adopt a tool with appropriate data storage and data use protocol, combined with appropriate guidance and training to promote ethical and responsible AI use to educators and students alike.^56^, ^57^ A vetted AI tool with an institutional/enterprise licence may offer added security and control of approved data usage, as well as allowing more equitable access to the tool amongst educators and learners.^56^

In conclusion, LLMs show satisfactory capabilities in answering traditional examination questions within the field of optometry and vision science, challenging traditional modes of assessment. Educational institutions are required to consider revising their assessment practices with the increasing proliferation and capabilities of GenAI tools, and implement frameworks and guidance to foster their ethical, responsible and effective use.

## AUTHOR CONTRIBUTIONS

**Nayuta Yoshioka:** Conceptualization (equal); data curation (lead); formal analysis (lead); investigation (lead); methodology (equal); resources (equal); supervision (lead); writing – original draft (lead); writing – review and editing (equal). **Vanessa Honson:** Conceptualization (equal); investigation (supporting); methodology (equal); resources (equal); writing – original draft (supporting); writing – review and editing (equal). **Revathy Mani:** Conceptualization (equal); methodology (equal); resources (equal); writing – original draft (supporting); writing – review and editing (equal). **Sharon Oberstein:** Conceptualization (equal); methodology (equal); resources (equal); writing – original draft (supporting); writing – review and editing (equal). **Kathleen Watt:** Conceptualization (equal); methodology (equal); resources (equal); writing – original draft (supporting); writing – review and editing (equal). **Vinod Maseedupally:** Conceptualization (equal); formal analysis (supporting); methodology (equal); resources (equal); writing – original draft (supporting); writing – review and editing (equal).

## CONFLICT OF INTEREST STATEMENT

The authors declare no conflicts of interest.

## FUNDING INFORMATION

No funding or grant was received for this research.

## Supporting information


Table S1:
