# Supplementary material for: Performance of ChatGPT on optometry and vision science exam questions
Source: Ophthalmic Physiol Opt. 2025 Jul 9;45(6):1376–88. doi: 10.1111/opo.13544 (PMC12357226; doi:10.1111/opo.13544)
Supplement: Supplementary file 1 — Table S1: [file OPO-45-1376-s001.docx]

### Supplementary Table S1

Full results from Tukey’s multiple comparison testing comparing the effect of the question type and models.

|  | Mean 1 | Mean 2 | Mean Diff. | 95% CI of difference | Adjusted P |
| --- | --- | --- | --- | --- | --- |
| D1: |  |  |  |  |  |
| GPT-3.5 vs. GPT-4 | 67.50 | 74.17 | -6.67 | -19.03 to 5.70 | 0.28 |
| GPT-3.5 vs. O1 | 67.50 | 98.33 | -30.83 | -49.81 to -11.85 | 0.008 |
| GPT-4 vs. O1 | 74.17 | 98.33 | -24.17 | -37.72 to -10.61 | 0.005 |
|  |  |  |  |  |  |
| C1: |  |  |  |  |  |
| GPT-3.5 vs. GPT-4 | 38.09 | 66.07 | -27.98 | -39.38 to -16.59 | 0.001 |
| GPT-3.5 vs. O1 | 38.09 | 87.78 | -49.69 | -60.44 to -38.93 | <0.0001 |
| GPT-4 vs. O1 | 66.07 | 87.78 | -21.70 | -35.61 to -7.80 | 0.008 |
|  |  |  |  |  |  |
| B1: |  |  |  |  |  |
| GPT-3.5 vs. GPT-4 | 64.69 | 70.97 | -6.28 | -19.44 to 6.87 | 0.35 |
| GPT-3.5 vs. O1 | 64.69 | 85.81 | -21.13 | -33.24 to -9.01 | 0.006 |
| GPT-4 vs. O1 | 70.97 | 85.81 | -14.84 | -22.71 to -6.98 | 0.004 |
|  |  |  |  |  |  |
| P1: |  |  |  |  |  |
| GPT-3.5 vs. GPT-4 | 52.14 | 55.00 | -2.86 | -17.18 to 11.46 | 0.85 |
| GPT-3.5 vs. O1 | 53.57 | 86.91 | -33.33 | -44.83 to -21.84 | 0.0005 |
| GPT-4 vs. O1 | 55.95 | 86.91 | -30.95 | -53.93 to -7.97 | 0.02 |
|  |  |  |  |  |  |
| P2: |  |  |  |  |  |
| GPT-3.5 vs. GPT-4 | 57.45 | 76.86 | -19.41 | -28.24 to -10.58 | 0.002 |
| GPT-3.5 vs. O1 | 57.45 | 88.82 | -31.37 | -42.00 to -20.74 | 0.0005 |
| GPT-4 vs. O1 | 76.86 | 88.82 | -11.96 | -18.77 to -5.16 | 0.005 |
|  |  |  |  |  |  |
| GPT-3.5: |  |  |  |  |  |
| D1 vs. C1 | 65.38 | 38.09 | 27.29 | 15.54 to 39.05 | <0.0001 |
| D1 vs. B1 | 65.38 | 64.69 | 0.70 | -10.65 to 12.05 | 0.99 |
| D1 vs. P1 | 65.38 | 52.14 | 13.24 | -1.91 to 28.39 | 0.10 |
| D1 vs. P2 | 65.38 | 57.45 | 7.93 | -4.02 to 19.89 | 0.30 |
| C1 vs. B1 | 38.09 | 64.69 | -26.59 | -37.24 to -15.95 | <0.0001 |
| C1 vs. P1 | 38.09 | 52.14 | -14.05 | -28.61 to 0.51 | 0.06 |
| C1 vs. P2 | 38.09 | 57.45 | -19.36 | -30.67 to -8.05 | 0.002 |
| B1 vs. P1 | 64.69 | 52.14 | 12.54 | -1.76 to 26.85 | 0.10 |
| B1 vs. P2 | 64.69 | 57.45 | 7.236 | -3.66 to 18.13 | 0.26 |
| P1 vs. P2 | 52.14 | 57.45 | -5.31 | -20.00 to 9.38 | 0.79 |
|  |  |  |  |  |  |
| GPT-4: |  |  |  |  |  |
| D1 vs. C1 | 74.17 | 66.07 | 8.09 | -7.49 to 23.67 | 0.47 |
| D1 vs. B1 | 74.17 | 70.97 | 3.20 | -10.23 to 16.63 | 0.92 |
| D1 vs. P1 | 74.17 | 55.00 | 19.17 | 4.70 to 33.63 | 0.008 |
| D1 vs. P2 | 74.17 | 76.86 | -2.70 | -15.55 to 10.16 | 0.94 |
| C1 vs. B1 | 66.07 | 70.97 | -4.90 | -18.82 to 9.03 | 0.76 |
| C1 vs. P1 | 66.07 | 55.00 | 11.07 | -3.80 to 25.94 | 0.19 |
| C1 vs. P2 | 66.07 | 76.86 | -10.79 | -24.22 to 2.65 | 0.12 |
| B1 vs. P1 | 70.97 | 55.00 | 15.97 | 3.71 to 28.23 | 0.009 |
| B1 vs. P2 | 70.97 | 76.86 | -5.89 | -15.11 to 3.32 | 0.27 |
| P1 vs. P2 | 55.00 | 76.86 | -21.86 | -33.04 to -10.68 | 0.0003 |
|  |  |  |  |  |  |
| O1: |  |  |  |  |  |
| D1 vs. C1 | 98.33 | 87.78 | 10.56 | 1.79 to 19.32 | 0.02 |
| D1 vs. B1 | 98.33 | 85.81 | 12.52 | 4.48 to 20.56 | 0.003 |
| D1 vs. P1 | 98.33 | 86.91 | 11.43 | -9.64 to 32.49 | 0.35 |
| D1 vs. P2 | 98.33 | 88.82 | 9.51 | 2.22 to 16.80 | 0.01 |
| C1 vs. B1 | 87.78 | 85.81 | 1.97 | -7.01 to 10.94 | 0.95 |
| C1 vs. P1 | 87.78 | 86.91 | 0.87 | -20.14 to 21.89 | 0.99 |
| C1 vs. P2 | 87.78 | 88.82 | -1.05 | -9.48 to 7.39 | 0.99 |
| B1 vs. P1 | 85.81 | 86.91 | -1.09 | -22.14 to 19.95 | 0.99 |
| B1 vs. P2 | 85.81 | 88.82 | -3.01 | -10.63 to 4.61 | 0.69 |
| P1 vs. P2 | 86.91 | 88.82 | -1.92 | -23.04 to 19.20 | 0.99 |
